# Mechanical Homogenisation of TPMS Architectures: A Comparison Between Finite Element and Mechanics of Structure Genome Approaches

**DOI:** 10.3390/ma18235357

**Published:** 2025-11-27

**Authors:** Sara Mouman, Yao Koutsawa, Lucas Binsfeld, Levent Kirkayak, Jieun Yang, Gaetano Giunta

**Affiliations:** 1 Luxembourg Institute of Science and Technology, 5 Avenue des Hauts-Fourneaux, L-4362 Esch-sur-Alzette, Luxembourg or sara.mouman@list.lu (S.M.); yao.koutsawa@list.lu (Y.K.); lucas.binsfeld@list.lu (L.B.);; 2 Faculty of Mechanical Engineering, Delft University of Technology, Mekelweg 2, 2628 CD Delft, The Netherlands; jieun.yang@tudelft.nl

**Keywords:** lattice materials, triply periodic minimal surfaces, numerical homogenisation, finite element analysis, mechanics of structure genome

## Abstract

This work presents a comparative study on the mechanical homogenisation of Triply Periodic Minimal Surface (TPMS) lattice structures in the linear elastic regime, which have attracted significant interest for their unique ability to combine lightweight design with tailored properties. The study investigates the effective mechanical behaviour of Representative Unit Cells (RUCs) generated using the open-source Python tool Microgen. Two homogenisation strategies are considered: (i) Finite Element (FE)-based homogenisation carried out in Abaqus, and (ii) the Mechanics of Structure Genome (MSG), a unified theory for multi-scale constitutive modelling, implemented in an in-house software tool. The comparison encompasses multiple TPMS topologies, including well-studied cases used for validation, namely gyroid and diamond, as well as less-explored ones, such as PMY and F-Rhombic Dodecahedron, to provide new insights. RUCs are analysed across relative densities ranging from 10 to 50%. Equivalent linear elastic properties (Young’s moduli, shear moduli, and Poisson’s ratios) are derived and compared to assess the consistency, accuracy, and computational efficiency of the two approaches. The results show that both methods yield effective properties with less than 1% difference between them, and less than 5% deviation from experimental data reported in the literature for the effective Young’s modulus. Furthermore, the anisotropy of each TPMS topology across the range of relative densities is examined through the directional distribution of Young’s moduli. The outcomes are expected to clarify the strengths and limitations of FE versus MSG in capturing the effective behaviour of architected cellular solids, thus supporting the selection of homogenisation strategies for the design of lattice-based lightweight structures.

## 1. Introduction

Metamaterials and architecture cellular solids based on Triply Periodic Minimal Surfaces (TPMSs) have attracted significant interest due to their unique ability to combine lightweight design with tailored mechanical, thermal, and acoustic properties. TPMSs were first described in 1865 by Schwarz [1], who introduced notable examples such as the Schwarz primitive and diamond surfaces. Subsequent developments were made by Neovius [2] in 1883, who proposed the Neovius surfaces, and Schoen [3] in 1970, who identified the gyroid and I-Wrapped Package (IWP) surfaces. TPMSs are found not only in various natural systems, such as butterfly wing scales and biological membranes, but also in artificial systems such as zeolite crystals [4]. These surfaces can be modelled using implicit mathematical equations, often represented by Fourier series, whose coefficients dictate both the surface topology and its mechanical behaviour [5]. Furthermore, TPMS architectures can be differentiated as sheet-based or solid-based structures: sheet-based TPMSs are generated by thickening the surface geometry, whereas solid-based TPMSs are created by solidifying the regions separated by the TPMS parametric function [6].

Triply Periodic Minimal Surfaces have emerged as promising candidates because of their attractive mechanical performance, particularly when compared to conventional lattice structures. They can also have tunable thermal properties, such as quasi-1D materials, which have charge density wave properties that define the behaviour of the material as temperature changes [7]. TPMSs have smooth surfaces and continuous periodic architectures that make them particularly suitable for additive manufacturing, including more advanced methods of manufacturing such as 4D printing [8], enabling structures with high stiffness-to-weight ratios [9], tunable energy absorption [10], and multifunctional performance [11]. These characteristics have already led to applications in aerospace, automotive, and biomedical engineering, ranging from lightweight sandwich cores [12] to impact-mitigation components [13] and bone scaffolds [14]. Additionally, TPMSs can overcome one of the primary limitations of truss-based lattices, namely the stress concentrations that occur at the joints, by offering better interconnectivity and reducing the likelihood of failure under higher loads [15,16].

To fully exploit the potential of TPMSs in design and optimisation, reliable prediction of effective mechanical properties is essential, as direct experimental testing of each topology and density level is often impractical and computationally expensive at the full-structure scale. The prediction of the effective mechanical behaviour of various TPMS topologies has already been explored by several authors, employing different methods for generating the topologies and performing the homogenisation. Refai et al. [17] employed the Finite Element Method (FEM) to study multiple geometries, including the gyroid, and reported that all showed an elastic response governed by cubic symmetry, except for the octahedral geometry, which displayed transverse isotropy. Ramirez et al. [18] also applied FEM-based homogenisation to primitive and gyroid structures at different relative densities and compared the results with those of array configurations of the same Representative Unit Cells (RUCs) obtained through numerical analyses. Zhang et al. [19] focused on hybrid TPMS structures, combinations of multiple topologies formed by Boolean union, with the objective of reducing structural anisotropy. By contrast, Liu et al. [20] adopted asymptotic homogenisation, implemented in MATLAB R2016b using voxel models, to study the primitive, IWP, and gyroid topologies. Despite the growing adoption of TPMS-based lattices, accurately and efficiently predicting their effective mechanical behaviour remains challenging. FEM homogenisation is widely used and provides detailed insight, but it often requires high computational effort, especially when exploring multiple relative densities and topologies.

In contrast, the Mechanics of Structure Genome (MSG) method [21] offers an alternative semi-analytical framework that can significantly reduce computational cost while preserving accuracy in capturing the essential physics of periodic heterogeneous materials. The MSG approach is conceptually derived from the Variational Asymptotic Method for Unit Cell Homogenisation (VAMUCH) but extends this framework by enabling the analysis of both periodic and non-periodic structures [22]. Alternatively, Fast Fourier Transform (FFT)-based homogenisation methods offer improved computational efficiency; however, they may sacrifice accuracy compared with the MSG approach, often leading to an underestimation of effective properties [23].

The novelty of this work lies in providing the first comprehensive comparison between FEM and MSG approaches for the mechanical homogenisation of TPMS structures in the linear elastic regime. This study clarifies the relative strengths, limitations, and application domains of both methods within the design workflow of architected metamaterials.

The paper is structured as follows. In Section 2, the modelling of TPMSs and RUCs is introduced, followed by the theoretical background of FEM-based RUC homogenisation and MSG-based homogenisation, along with a comparison between the two methods. Section 3 presents the homogenisation results obtained for the different TPMS structures, including the effective elastic properties, as well as the distribution of the directional Young’s modulus. These results are then used to compare the two approaches in terms of their strengths and weaknesses. Finally, Section 4 summarises the main findings of the work.

## 2. Methodology

In this section, the approach used for generating the TPMS and RUC structures is presented, together with a description of the homogenisation methods employed, namely FEM-based RUC homogenisation and MSG-based homogenisation.

### 2.1. Generation of TPMS Geometry

To generate TPMS models for FEM or MSG analysis, different methods can be followed. One option is to create isosurfaces using parametric equations in software such as MATLAB [24] or Surface Evolver [25] among many, then add thickness in a CAD program to obtain a solid model [26]. The geometry can subsequently be meshed in tools like Hypermesh [27] or Gmsh 4.12 [28]. Another alternative is to use dedicated software for modelling TPMS structures, such as MSLattice [29]. With MSLattice, RUCs of various TPMS topologies can be created with different sizes, relative densities, and resolutions, as well as with customised shapes and functional grading [19]. Both sheet and solid TPMSs can be generated. The output files are in STL format; therefore, additional software is required to generate solid models for further analysis.

**Figure 1:**
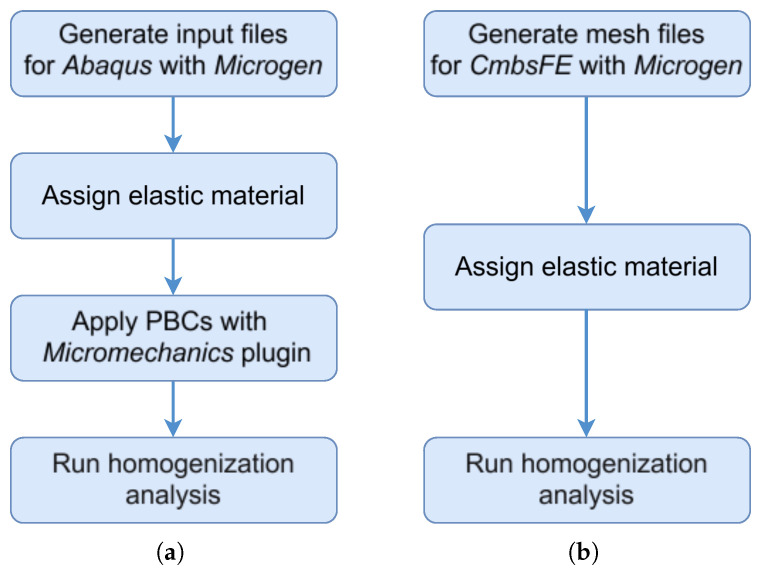
Homogenisation analysis workflow: (**a**) FEM-based analysis in Abaqus. (**b**) MSG-based analysis in the in-house code CmbsFE.

A more streamlined alternative, used in this work, is provided by the Python library Microgen 1.3.2 [30]. This library enables the modelling of a wide range of RUCs, from strut-based unit cells to general TPMS topologies. Furthermore, it is possible to extend the original code to model any type of TPMS using parametric equations. Similarly to MSLattice, Microgen is not limited to unit cells, but also supports the generation of array configurations and spherical shapes. It offers flexibility in output formats, including STL files, mesh files, and even input files that can be used directly in Abaqus 2022, removing the need for intermediate software. An important aspect when performing FEM homogenisation with Periodic Boundary Conditions (PBCs) is to ensure that the mesh is periodic such that these boundary conditions are applied correctly and the results remain reliable. Microgen addresses this requirement by offering a function to create periodic meshes through Gmsh. The general workflow followed to carry the mechanical homogenisation analysis for both approaches is illustrated in Figure 1.

Depending on the software used for the RUC generation, it is possible to either have as an input the thickness of the RUC or its relative density. In most of the literature reviewed, the latter is more commonly used, as is also the case for Microgen. Furthermore, our tool assessment showed that the geometry created is not guaranteed to be identical even when the same TPMS topology is selected in Microgen or MSLattice. There might be differences in the thickness spatial distribution due to how the material is distributed based on the algorithms used to create the geometry and the calculations followed to calculate the relative density.

### 2.2. FEM-Based RUC Homogenisation

To determine the effective mechanical properties of the TPMS architectures, this study employs a computational homogenisation scheme based on the finite element analysis of a Representative Unit Cell. This approach, often termed “virtual testing”, is a robust and widely accepted method for characterising complex microstructures by simulating their response under well-defined boundary conditions [31]. The fundamental principle is to replace the complex, heterogeneous TPMS lattice with an equivalent (or effective) homogeneous material, whose constitutive behaviour can be described by a constant, effective material stiffness tensor $C_{ijkl}^{*}$.

The relationship between the volume-averaged (macroscopic) stress tensor ${\bar{\sigma }}_{ij}$ and strain tensor ${\bar{\epsilon }}_{ij}$ for the equivalent homogeneous material is governed by the generalised Hooke’s law:

(1) $${\bar{\sigma }}_{ij}=C_{ijkl}^{*}{\bar{\epsilon }}_{kl}.$$

Within the RUC, the local stress field $\sigma_{ij}$ must satisfy the static equilibrium equation, which, assuming the absence of body forces, reads [31]:

(2) $$\sigma_{ij,j}=0,$$

where an index preceded by a comma stands for a derivative with respect to that index.

Combining this with the local constitutive relation and the strain-displacement kinematics, the problem is formulated in terms of the displacement field $u_{i}$ as a well-posed boundary value problem.The objective of the RUC analysis is to solve for the local stress and strain fields resulting from a set of prescribed macroscopic strains and then, compute the corresponding macroscopic stresses to populate the effective stiffness matrix $C_{ijkl}^{*}$.

For periodic materials such as TPMS lattices, the choice of boundary conditions is critical. Periodic Boundary Conditions are considered the most appropriate as they accurately represent a unit cell’s kinematic constraints within an infinite lattice and satisfy the Hill-Mandel condition of macro-homogeneity, ensuring energetic equivalence between the micro- and macro-scales. PBCs enforce that the displacement fluctuations on opposite faces of the RUC are equal, and that the tractions are equal and opposite. The displacement $u_{i}$ at a point $y_{j}$ within the RUC is decomposed into a macroscopic part and a fluctuating part $\chi_{i}$ [31]:

(3) $$u_{i}={\bar{\epsilon }}_{ij}y_{j}+\chi_{i}.$$

The periodicity constraint is applied to the fluctuation field, requiring that it have the same value at corresponding points on opposite faces of the RUC cube (e.g., faces at $y_{k}=Y_{k}^{+}$ and $y_{k}=Y_{k}^{-}$):

(4) $$\chi_{i}\left(y_{j}^{+}\right)=\chi_{i}\left(y_{j}^{-}\right).$$

In the Abaqus/CAE environment, these conditions are implemented by applying constraint equations that link the Degrees of Freedom (DOFs) of corresponding node pairs on opposite faces of the TPMS unit cell [32].

The effective stiffness matrix is computed by conducting a series of six independent static linear elastic simulations on the high-fidelity finite element mesh of the TPMS RUC. For each simulation, a distinct unit macroscopic strain component is prescribed, while all other components are set to zero. The six fundamental load cases correspond to three normal strains (${\bar{\epsilon }}_{11},{\bar{\epsilon }}_{22},{\bar{\epsilon }}_{33}$) and three shear strains (${\bar{\gamma }}_{12},{\bar{\gamma }}_{13},{\bar{\gamma }}_{23}$).

For each of the six analyses, the following steps are performed:

1. A single component of the macroscopic strain tensor ${\bar{\epsilon }}_{ij}$ is set to unity (e.g., ${\bar{\epsilon }}_{11}=1$), and all others are set to zero. This macroscopic strain is enforced on the RUC via the PBCs.
2. A static analysis is performed in Abaqus to solve for the resulting local stress field $\sigma_{ij}$ throughout the entire volume *V* of the RUC.
3. The macroscopic stress tensor ${\bar{\sigma }}_{ij}$ is calculated by volume-averaging the local stress field [31]:
  (5) $${\bar{\sigma }}_{ij}=\langle \sigma_{ij}\rangle =\frac{1}{V}\int_{V}\sigma_{ij}dV.$$
4. The resulting six-component vector of volume-averaged macroscopic stresses $\{{\bar{\sigma }}_{11}$, ${\bar{\sigma }}_{22}$, ${\bar{\sigma }}_{33}$, ${\bar{\sigma }}_{12}$, ${\bar{\sigma }}_{13}$, ${\bar{\sigma }}_{23}{\}}^{T}$ forms the corresponding column of the 6×6 effective stiffness matrix.

By repeating this procedure for all six unit strain cases, the complete anisotropic stiffness matrix for the TPMS architecture is systematically populated.

### 2.3. Mechanics of Structure Genome-Based Homogenisation

To determine the effective mechanical properties of TPMS architectures, this study also employs the Mechanics of Structure Genome methodology. MSG is a unified multi-scale constitutive modelling approach that is particularly well-suited for periodic heterogeneous materials [21]. The core of the methodology is the Structure Genome (SG), defined as the smallest mathematical building block that contains all the necessary constitutive information of the structure, analogous to how a biological genome contains the information for an organism’s development.

For a material with three-dimensional heterogeneity, such as a TPMS lattice, the SG is a three-dimensional repeating unit cell, serving a similar role to the conventional RUC [21,33,34]. However, the strength of MSG lies in its rigorous theoretical foundation, which is based on the principle of minimum information loss. This principle states that the homogenised model should be constructed by minimizing the discrepancy in strain energy between the original heterogeneous model and the effective homogeneous model.

The formulation begins by decomposing the displacement field $u_{i}$ within the SG into a macroscopic homogenised component ${\bar{u}}_{i}$ and a fluctuating component $\chi_{i}$, which captures the local microstructural variations. This kinematic relationship is expressed as follows:

(6) $$u_{i}\left(x,y\right)={\bar{u}}_{i}\left(x\right)+\chi_{i}\left(x,y\right),$$

where x and y represent the macroscopic and microscopic coordinates, respectively. The macroscopic displacement ${\bar{u}}_{i}$ is defined as the volume average of the local displacement field, ${\bar{u}}_{i}=\langle u_{i}\rangle$. This definition imposes a zero-average constraint on the fluctuation function, i.e., $\langle \chi_{i}\rangle =0$.

Following this kinematic assumption, the local strain field $\epsilon_{ij}$ can be related to the macroscopic strain ${\bar{\epsilon }}_{ij}$ and the gradient of the fluctuation function. Neglecting higher-order terms, this relationship is given by:

(7) $$\epsilon_{ij}\left(x,y\right)={\bar{\epsilon }}_{ij}\left(x\right)+\chi_{i,j},$$

where $\chi_{i,j}$ denotes the symmetric part of the gradient of the fluctuation function. To solve for the unknown fluctuation function $\chi_{i}$, MSG minimizes the strain energy of the original model over the SG domain, subject to the relevant constraints. The variational problem leads to the following Euler-Lagrange equation, which governs the response of the SG [21,33]:

(8) $${\left[C_{ijkl}\left({\bar{\epsilon }}_{kl}+\chi_{k,l}\right)\right]}_{,j}=0.$$

Here, $C_{ijkl}$ is the local, spatially varying stiffness tensor of the constituent material within the SG. For TPMS architectures, which are periodic structures, the fluctuation function $\chi_{i}$ must also be periodic. This governing equation can be solved numerically (e.g., using the finite element method) to find $\chi_{i}$ in terms of the prescribed macroscopic strains ${\bar{\epsilon }}_{kl}$.

The solution can be expressed symbolically as $\chi_{k}=H_{k}^{mn}{\bar{\epsilon }}_{mn}$, where $H_{k}^{mn}$ is an influence function tensor that links the macroscopic strains to the local displacement fluctuations [21,33]. Once determined, the effective stiffness tensor $C_{ijkl}^{*}$ for the homogenised TPMS structure is computed by volume averaging the strain energy density, yielding the final expression:

(9) $$C_{ijkl}^{*}=\langle C_{ijkl}+C_{ijmn}H_{m,n}^{kl}\rangle .$$

This formulation provides a direct and computationally efficient pathway to calculate the full three-dimensional anisotropic stiffness matrix of the TPMS architecture from a single analysis of its fundamental building block, the SG.

### 2.4. Comparison of RUC Analysis and MSG

While both the FE-based RUC analysis and the MSG are powerful tools for mechanical homogenisation, they are founded on different theoretical principles and exhibit significant differences in computational implementation and scalability. This section provides a comparative analysis of the two approaches, contextualising their application to the multi-scale modelling of TPMS architectures.

#### 2.4.1. Theoretical Equivalence for 3D Periodic Media

For the specific case of a three-dimensional, periodic microstructure, such as the TPMS lattices studied in this work, the underlying governing equations for RUC analysis with PBCs and MSG are identical. It should be noted that this equivalence can be considered valid only for linear elastic models with perfect periodicity. Both methods ultimately solve for a displacement fluctuation field $\chi_{i}$ that satisfies the same equilibrium equation given in Equation (8) within the unit cell [31]. Consequently, when applied to the same three-dimensional unit cell with PBCs, both RUC analysis and MSG are expected to yield the exact same results for effective properties and local fields, provided the numerical discretisation is equivalent.

The primary distinction, therefore, lies not in the final result for this class of problems, but in the efficiency and elegance of the computational workflow.

#### 2.4.2. Computational Implementation and Efficiency

A significant divergence between the two methods emerges from their computational implementation. A standard RUC analysis performed in commercial FE software like Abaqus typically requires setting up and solving six independent boundary value problems to populate the 6×6 stiffness matrix. The application of PBCs via coupled equation constraints affects the global stiffness matrix of the FE model, necessitating a full re-factorisation for each of the six unit strain load cases.

In contrast, the MSG framework is structured to be algorithmically more efficient. Its implementation solves the problem by factorising the linear system’s coefficient matrix only once. The six load cases, corresponding to the six unit macroscopic strains, are then treated as multiple right-hand sides. This allows for the solution to be obtained with one factorisation followed by six back-substitutions, a process that is more efficient numerically. This makes the MSG approach theoretically five to six times more efficient than a conventional RUC analysis for computing the complete stiffness matrix. However, a direct comparison between the FEM and MSG approaches cannot be made, as the software used to perform the two analyses differs significantly in implementation. The FEM solution is obtained using Abaqus, a commercial code that has been extensively optimised to minimise computational time. In contrast, the MSG homogenisation is carried out with an in-house implementation of the MSG framework, developed primarily to demonstrate the methodology rather than to optimise computational performance in terms of memory usage and other efficiency-related aspects.

#### 2.4.3. Fundamental Advantages of the MSG Framework

Beyond computational speed-up for three-dimensional problems, MSG holds fundamental advantages that establish it as a more versatile and scalable multi-scale modelling theory.

Variational Foundation: MSG is derived directly from a variational statement seeking to minimise the information loss (in terms of strain energy) between the original and homogenised models. RUC analysis, while satisfying the Hill-Mandel condition, is typically formulated based on the strong form of the boundary value problem.This variational foundation gives MSG a more rigorous mathematical underpinning.

Dimensional Scalability: The most powerful feature of MSG is its ability to use a Structure Genome of the lowest possible dimensionality to characterise the material. For instance, to find the complete three-dimensional effective properties of a laminated composite (one-dimensional heterogeneity) or a continuous fibre-reinforced composite (two-dimensional heterogeneity), MSG can perform the analysis on a one- or two-dimensional SG, respectively. This drastically reduces the computational cost by avoiding the need to mesh and solve a full 3D domain. A conventional RUC analysis, in contrast, would still require a full three-dimensional RUC to extract its properties, making it unnecessarily expensive for such materials.

While the TPMS architectures in this study possess three-dimensional heterogeneity and, thus, require a three-dimensional SG, this inherent scalability makes MSG a superior general-purpose tool for the multi-scale analysis of a wider class of architected and composite materials, offering unparalleled efficiency without sacrificing accuracy.

## 3. Numerical Results and Discussion

To compare the FEM-based RUC homogenisation method with the MSG approach, a comprehensive study was conducted by analysing various TPMS topologies and predicting their effective elastic properties, with the structures being: gyroid, diamond, PMY and F-Rhombic Dodecahedron (F-RD). The FEM simulations were performed using Abaqus, with PBCs applied via the Micromechanics plug-in. For the MSG approach, the in-house C++ code CmbsFE, previously employed in Koutsawa et al. [33,34], was extended in this work to investigate the elastic behaviour of TPMS structures.

Each TPMS topology was studied over a range of relative densities from 10% to 50% to investigate very thin as well as more bulky configurations. From a manufacturing standpoint, it should be noted that structures with relative densities below approximately 20% can become mechanically unstable or even disconnected, depending on the surface topology and the method used to generate the geometry.

To ensure the reliability of the results, two configurations from previously published works were first reproduced to validate the methods used in this study. These configurations are presented in Table 1, together with the topology of the TPMS structure, the relative density of the RUC $\bar{\rho }$ (ratio between the effective volume of the TPMS RUC and that of the RUC bounding box), its size, and the base material properties (Young’s modulus $E_{s}$, shear modulus $G_{s}$ and Poisson’s ratio $\nu_{s}$).

**Figure 2:**
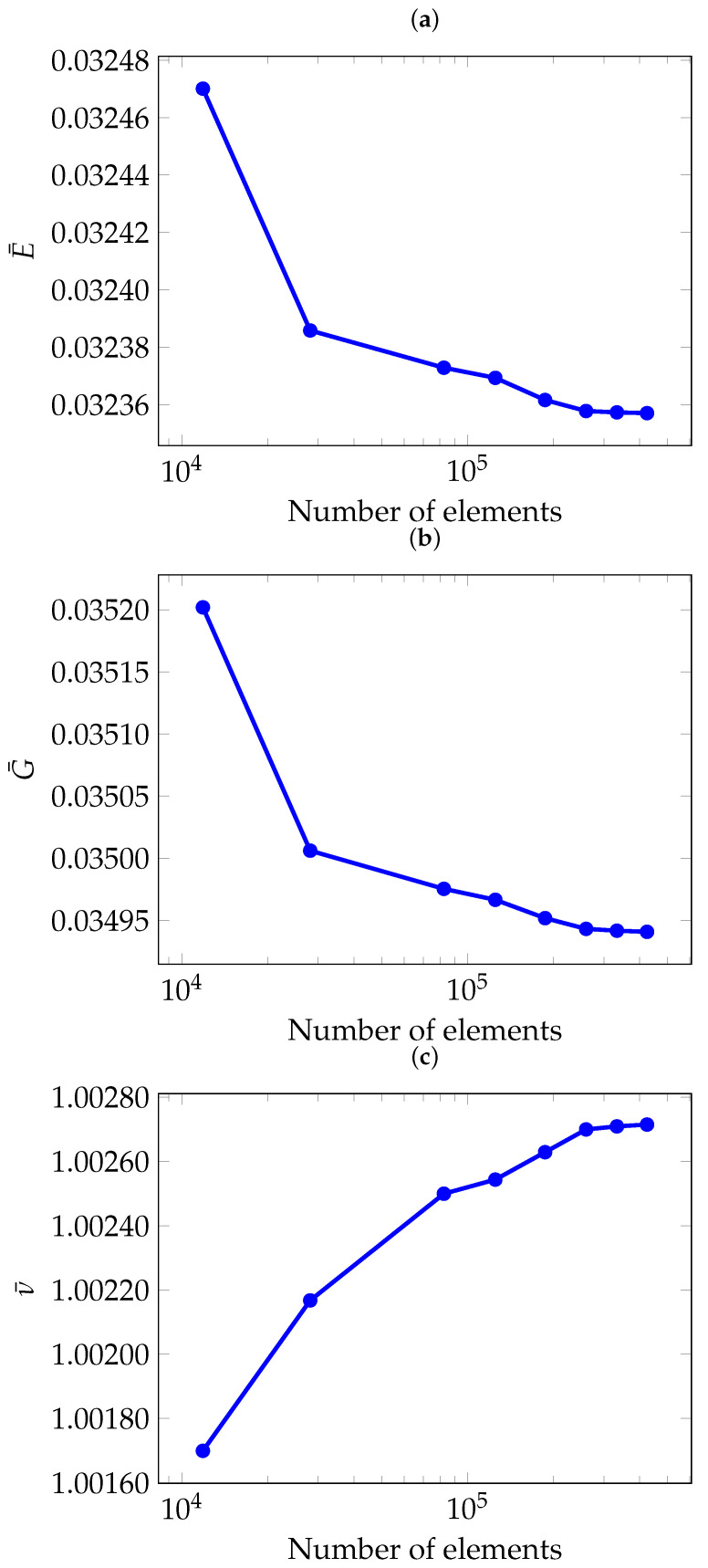
Convergence of effective elastic properties with respect to the number of elements: (**a**) Normalised Young’s modulus $\bar{E}$, (**b**) Normalised shear modulus $\bar{G}$, (**c**) Normalised Poisson’s ratio $\bar{\nu }$.

For all analysed models, a quadratic tetrahedral mesh was employed. A convergence study was conducted to ensure that the mesh resolution was sufficient to accurately estimate the effective properties. Figure 2 presents the convergence study for a 5 mm gyroid cell with 10 % relative density using the FEM method. Although the study was performed for both homogenisation approaches, only the FEM results are shown here to avoid redundancy. As can be seen, the results begin to converge even with relatively coarse meshes of 30,000 elements, achieving agreement within three significant figures. To ensure reliable predictions for all configurations, however, meshes of at least 170,000 elements were used in this study, corresponding to a minimum element size of 0.3 mm.

The effective elastic properties are normalised using the properties of the elastic material used to characterise the TPMS structures. In doing so, the properties are made independent of the bulk material used. The normalised properties are defined as follows:

(10) $$\begin{array}{ccc} \bar{E}=\frac{E_{i}^{*}}{E_{s}}, & \bar{G}=\frac{G_{ij}^{*}}{G_{s}}, & \bar{\nu }=\frac{\nu_{ij}^{*}}{\nu_{s}}, \end{array}$$

where $E_{i}$, $G_{ij}$, and $\nu_{ij}$ are respectively the effective Young’s modulus, shear modulus, and Poisson’s ratio.

### 3.1. Gyroid TPMS

**Figure 3:**
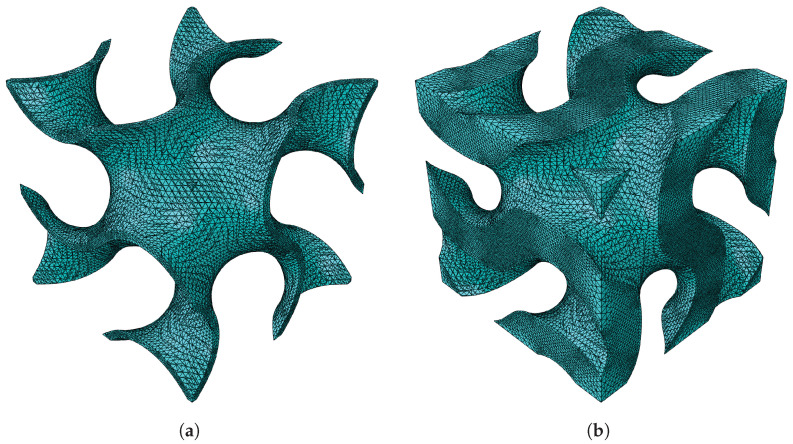
Gyroid RUC at minimum and maximum relative densities: (**a**) $\bar{\rho }=0.1$. (**b**) $\bar{\rho }=0.5$.

The first topology observed is the gyroid shape. The first configuration given in Table 1 is followed to validate the results obtained using FEM and MSG. For better visualisation, the gyroid RUCs are shown in Figure 3.

The normalised effective elastic properties are presented in Table 2 for the minimum and maximum relative densities analysed.

**Figure 4:**
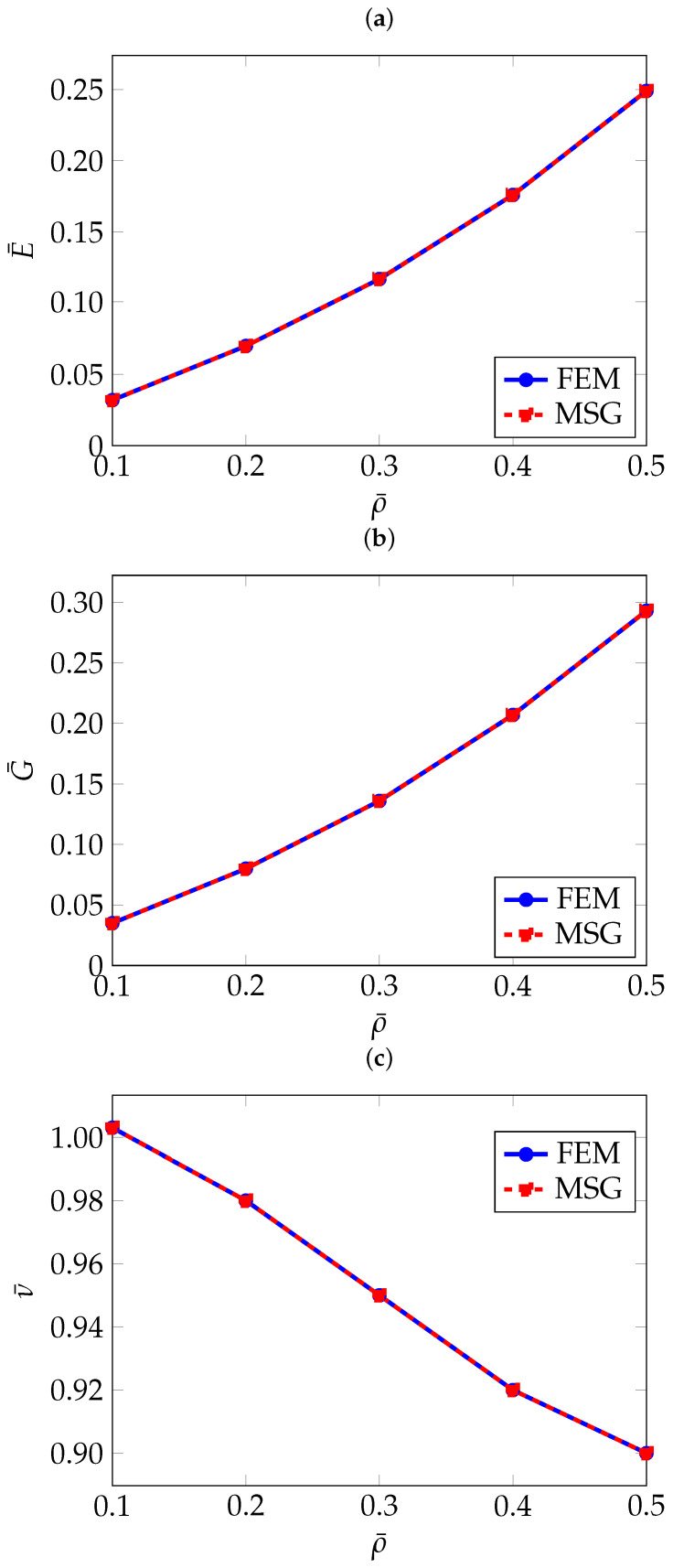
Normalised effective elastic properties of gyroid TPMS as a function of relative density: (**a**) Normalised effective Young’s modulus $\bar{E}$, (**b**) Normalised effective shear modulus $\bar{G}$, (**c**) Normalised effective Poisson’s ratio $\bar{\nu }$.

As can be seen in Table 2, the FEM and MSG-based results match closely. The effect of the relative density on the effective elastic properties can be better seen in Figure 4.

As it can be expected, the higher the relative density, and therefore a higher thickness, the higher both Young’s and shear moduli. On the other hand, the Poisson’s ratio exhibits an apparently opposite trend. Further analysis of RUCs with a relative density greater than 50% revealed that the Poisson’s ratio increases, approaching the value of the original solid material. This behaviour is expected, as higher relative density means the RUC becomes increasingly similar to the bulk material.

**Figure 5:**
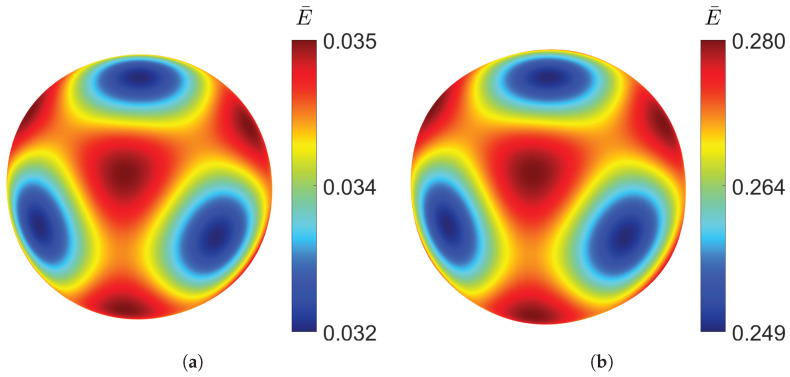
Young’s modulus distribution of gyroid TPMS at different relative densities: (**a**) $\bar{\rho }=0.1$. (**b**) $\bar{\rho }=0.5$.

The spatial distribution of the Young’s modulus can be determined from the effective stiffness tensor $C_{ijkl}^{*}$ obtained through homogenisation using the tool developed by Dong [35]. Figure 5 shows the directional distribution of the Young’s modulus for the gyroid structure for a relative density as low as 10% and as high as 50%.

It can be observed that even at low density, the distribution closely approaches a spherical shape, indicating a high degree of isotropy. Increasing the relative density does not produce significant changes in the distribution of the elastic modulus, demonstrating the structural stability. Although the variation is minimal, the gyroid is slightly stiffer along the principal axes than along the diagonal directions. The cubic symmetry, characteristic of TPMS structures, can also be clearly observed.

### 3.2. Diamond TPMS

**Figure 6:**
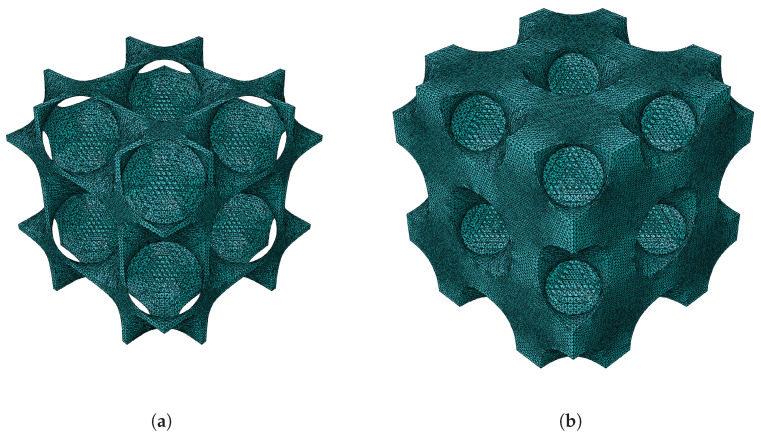
Diamond RUC at minimum and maximum relative densities: (**a**) $\bar{\rho }=0.1$. (**b**) $\bar{\rho }=0.5$.

For the diamond shape, the second configuration presented in Table 1 is considered. Figure 6 show the diamond RUCs at the lower and highest considered relative densities.

Table 3 presents the computed equivalent properties, together with the results reported by Zhang et al. [19] for a relative density of 30%. The latter include both numerical and experimental data, which are used for validation. As shown, all the simulation results are in close agreement. Regarding the experimental results, the second experiment yields similar values to the simulation results, while the first shows a deviation of approximately less than 5%, still indicating a high level of accuracy.

**Figure 7:**
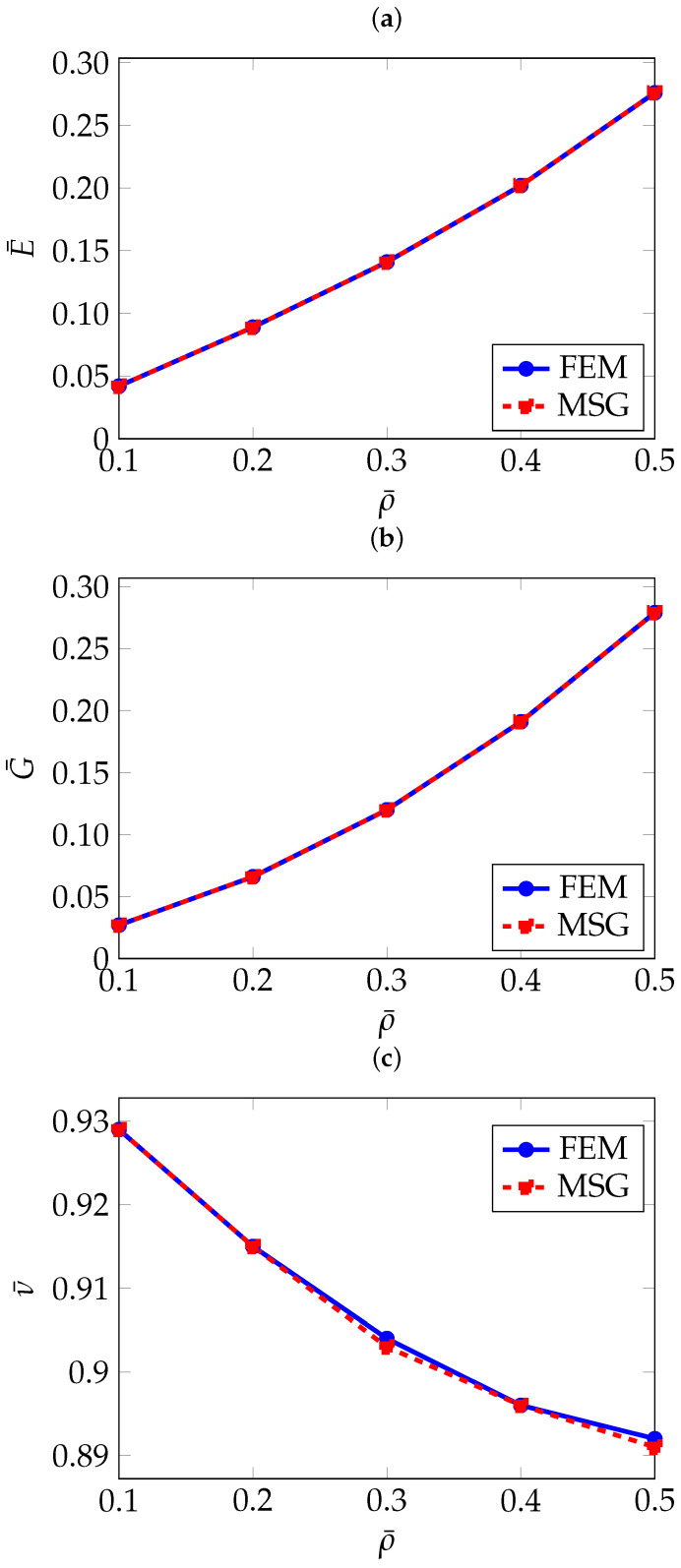
Normalised effective elastic properties of the diamond structure as a function of relative density: (**a**) Normalised effective Young’s modulus $\bar{E}$, (**b**) Normalised effective shear modulus $\bar{G}$, (**c**) Normalised effective Poisson’s ratio $\bar{\nu }$.

In Figure 7, the normalised effective elastic properties as a function of the relative density are given.

**Figure 8:**
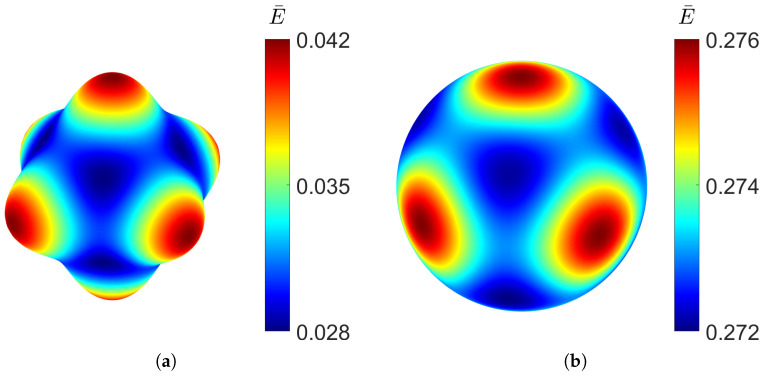
Young’s modulus distribution of diamond TPMS at different relative densities: (**a**) $\bar{\rho }=0.1$. (**b**) $\bar{\rho }=0.5$.

As observed for the gyroid structure, the diamond also shows an increase in Young’s modulus and shear modulus with higher relative density, and the opposite trend for the Poisson’s ratio. With a large difference between the elastic Young’s modulus along the principal axes and the diagonal directions, the diamond topology shows high levels of anisotropy at low densities, which can be observed in Figure 8.

As the volume of the material increases, the diamond TPMS behaviour becomes more isotropic, as shown by a spherical distribution for the RUC at relative density $\bar{\rho }=0.5$.

### 3.3. PMY

**Figure 9:**
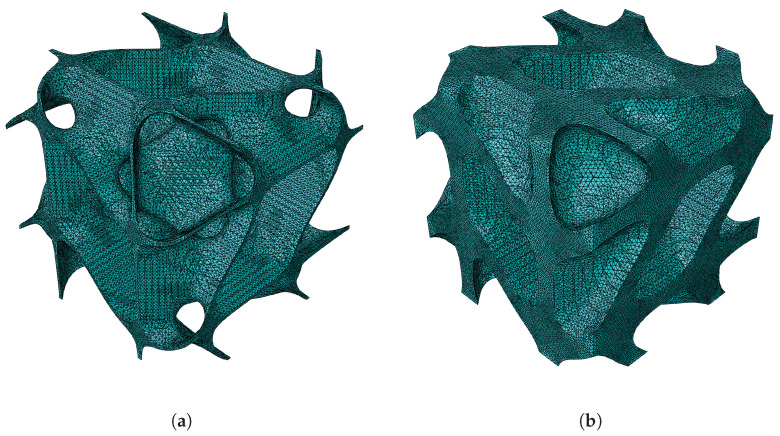
PMY RUC at minimum and maximum relative densities: (**a**) $\bar{\rho }=0.1$. (**b**) $\bar{\rho }=0.5$.

The effective elastic properties of the PMY structure are also investigated, where the geometrical parameters and base material properties of the second case in Table 1 are used. The PMY RUCs considered in this study are illustrated in Figure 9.

**Figure 10:**
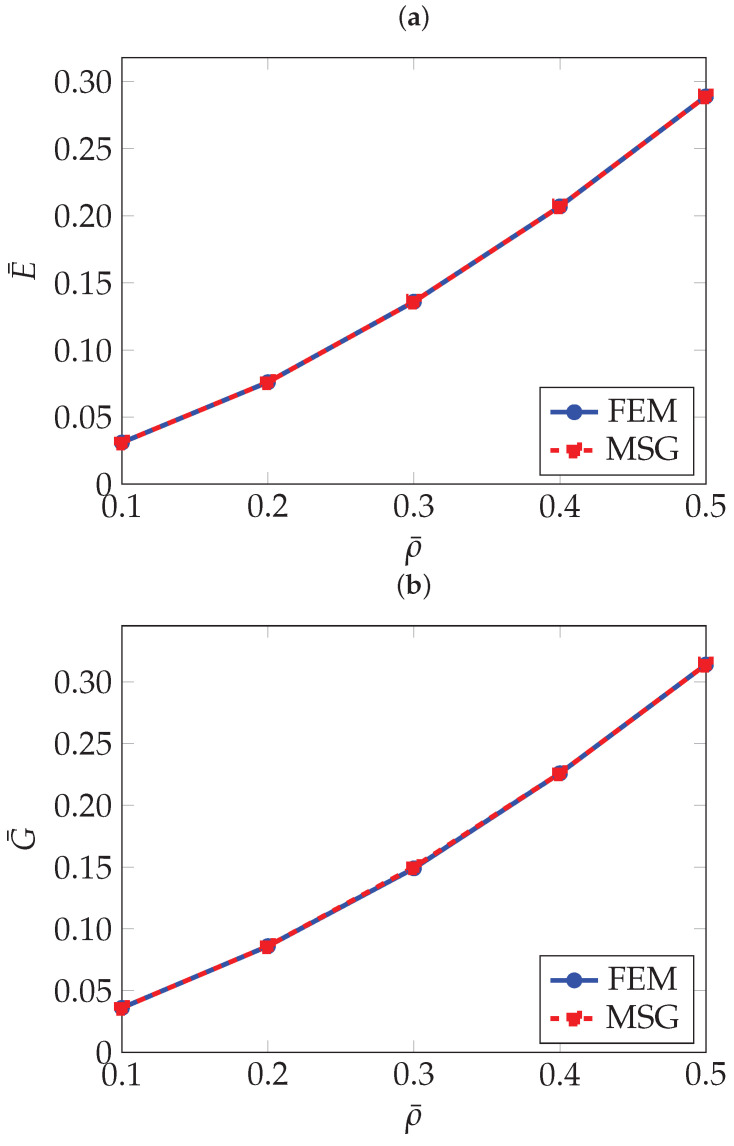

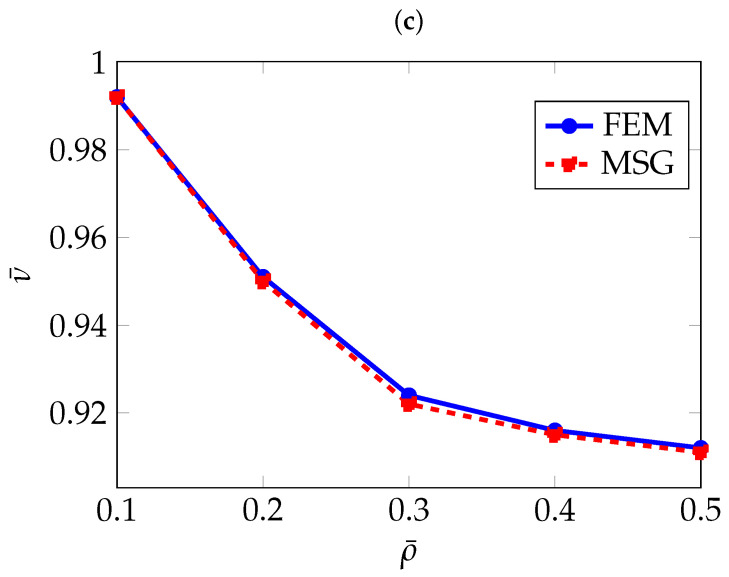
Normalised effective elastic properties of PMY as a function of relative density: (**a**) Normalised effective Young’s modulus $\bar{E}$, (**b**) Normalised effective shear modulus $\bar{G}$, (**c**) Normalised effective Poisson’s ratio $\bar{\nu }$.

To the author’s knowledge, this topology has not yet been widely investigated, therefore, the comparison is limited to the results obtained using the FEM and MSG methods. As shown in Table 4, which presents the homogenisation results, and in Figure 10, where these results are plotted as a function of relative density, a strong agreement is observed between the two methods.

**Figure 11:**
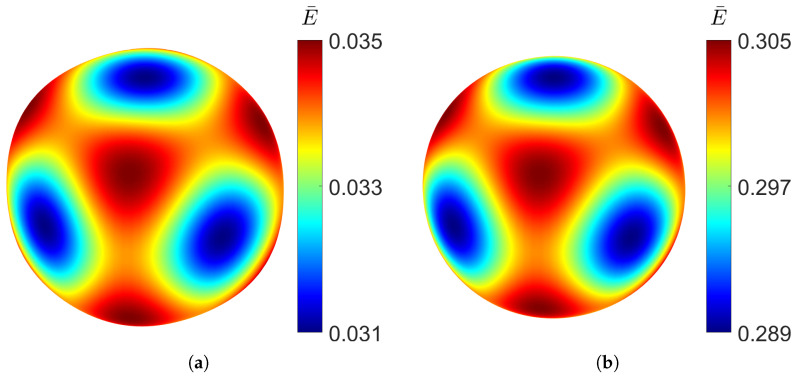
Elastic Young’s modulus distribution of PMY TPMS at different relative densities, RUC at: (**a**) $\bar{\rho }=0.1$ and (**b**) $\bar{\rho }=0.5$.

Similar to the gyroid shape, the PMY structure also shows high isotropy at low densities, as can be seen in Figure 11, where the directional Young’s modulus distribution is visualised.

However, the stiffness distribution shows that the diagonal directional Young’s modulus is the highest, whereas in the *x*, *y*, and *z* directions of the RUC, it is the lowest.

### 3.4. F-Rhombic Dodecahedron (F-RD)

**Figure 12:**
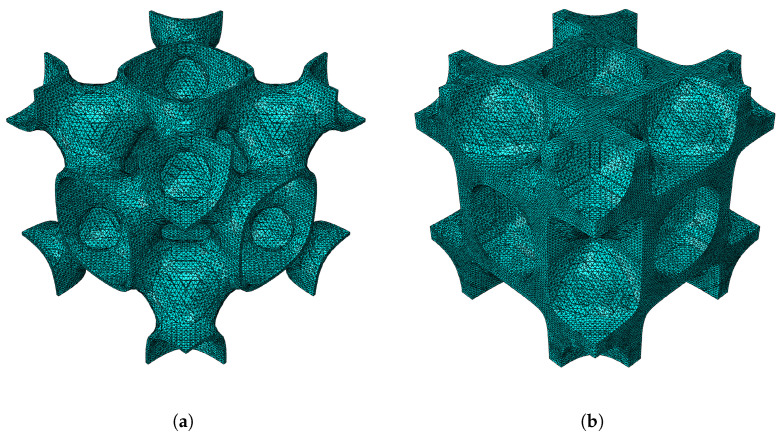
F-RD RUC at minimum and maximum relative densities: (**a**) $\bar{\rho }=0.1$, (**b**) $\bar{\rho }=0.5$.

For the F-RD shape, once again the second case properties in Table 1 are used. A visualisation of the RUCs investigated is given in Figure 12.

**Figure 13:**
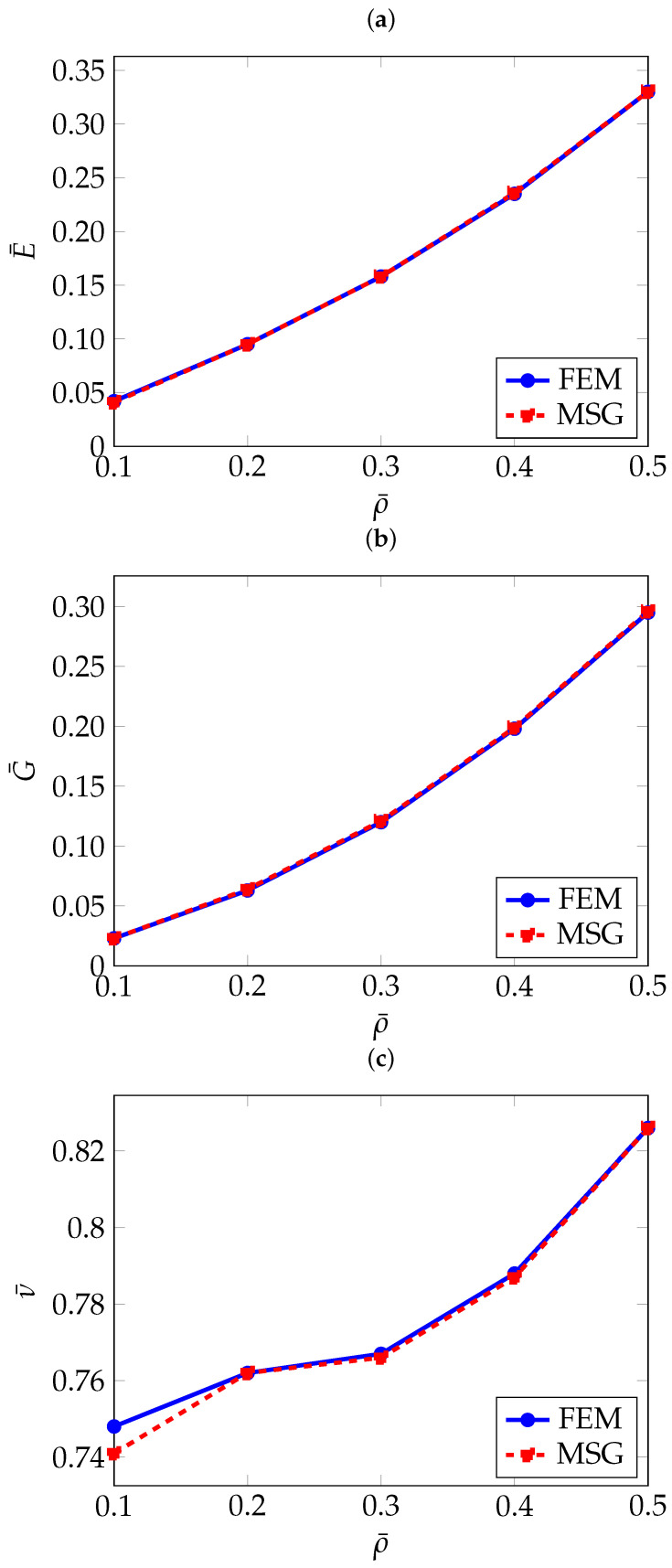
Normalised effective elastic properties of F-RD as a function of relative density: (**a**) Normalised effective Young’s modulus $\bar{E}$, (**b**) Normalised effective shear modulus $\bar{G}$, (**c**) Normalised effective Poisson’s ratio $\bar{\nu }$.

As with the previously analysed TPMS structures, the FEM and MSG results show good agreement, as illustrated in Table 5 and Figure 13, where the homogenisation results are plotted against relative density. A slight discrepancy can be observed in the Poisson’s ratio for the RUCs at a relative density of 10%, amounting to only 1.3%. This difference appears more pronounced due to the scale used to present the results.

**Figure 14:**
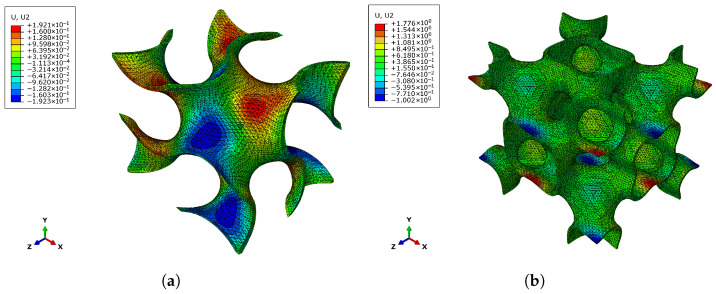
Transverse displacement [mm] along *y* direction while applying a deformation along *x* direction at relative density of 10%: (**a**) Gyroid and (**b**) F-RD.

However, unlike all the structures considered so far, the F-RD shape shows a positive trend with respect to a higher relative density not only with the elastic Young’s modulus and shear modulus, but also the Poisson’s ratio. This discrepancy can be better explained looking at the deformation mechanisms of the structures. An example is given in Figure 14, comparing the transverse displacement along *y* while applying a deformation along *x* for both gyroid and F-RD at 10% relative density. The figure clearly shows that the transverse contraction is very small and localised in the case of the F-RD topology, resulting in the Poisson’s ratio monotonically increasing with the relative density. On the other hand, the gyroid topology presents a much more widespread transverse contraction.

**Figure 15:**
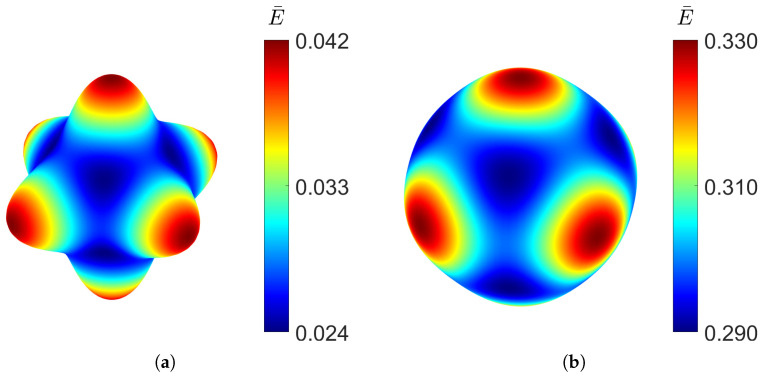
Elastic Young’s modulus distribution of F-RD TPMS at different relative densities, RUC at: (**a**) $\bar{\rho }=0.1$ and (**b**) $\bar{\rho }=0.5$.

Looking at the directional distribution of the Young’s modulus in Figure 15, the F-RD structure shows the highest anisotropy, having the biggest difference between the minimum and maximum Young’s modulus at low density. However, at high densities, the stiffness distribution becomes more uniform. Like the diamond structure, the F-RD is the stiffest along the coordinate axes.

## 4. Conclusions

In this paper, a study was conducted to compare the mechanical homogenisation of Triply Periodic Minimal Surfaces in the linear elastic regime, using two different methods: the Finite Element Method and the Mechanics of Structure Genome. The homogenisation analysis was focused on the Representative Unit Cells of four TPMS topologies: gyroid, diamond, PMY, and F-Rhombic Dodecahedron (F-RD). The first two were chosen as they have already been explored extensively in literature, allowing for a validation of the results obtained in this paper. The last two topologies were selected due to the lack of information on their effective elastic properties, helping to fill this gap in the literature.

The main parameter that was varied when studying the mechanical behaviour of the TPMS topologies was the relative density, in a range between 10% and 50%. In all the different TPMS configurations presented in this paper, both the FEM and MSG methods have produced results that match very closely. The simulation results from the two approaches differ by less than 1%, and when compared with available experimental data, the deviation in effective Young’s modulus is less than 5%, indicating good predictive accuracy.

Through the analysis of the linear elastic effective properties, it has been shown that all TPMSs have a higher Young’s modulus and shear modulus with increasing relative density. The opposite trend can be seen with the Poisson’s ratio for all TPMSs apart from the F-RD structure. The directional distribution of the Young’s modulus was also investigated with respect to relative density. It was seen that at low densities, TPMS structures can show different levels of anisotropy based on the topology chosen. The gyroid and the PMY structures show a spherical distribution of the stiffness, indicating high isotropy. On the other hand, the diamond and the F-RD are highly anisotropic at low densities. However, for all the shapes, as the relative density increases, the stiffness distribution becomes more isotropic, approaching that of a sphere.

With the results obtained, it was shown that the MSG method can reliably predict the effective elastic properties, reproducing the same values as those obtained with the FEM approach. It achieves this while providing intrinsic computational advantages: unlike the FEM-based RUC analysis, which requires multiple system factorisations to compute the full stiffness matrix, the MSG method performs a single factorisation followed by multiple back-substitutions.

This study focused on the homogenisation of mechanical properties; however, the approaches presented were limited to the linear elastic regime. Future work could extend these approaches to the study of effective thermo-mechanical and viscoelastic properties of TPMS structures. Finally, this work focused only on the collection of simulations results; carrying out an experimental study would provide valuable insight into how accurately these methods predict the real-world mechanical behaviour of architected metamaterials.

## Figures and Tables

**Table 1:** Configurations from the literature used for validation.

Source	TPMS	$\bar{\rho }$	RUC Size [mm]	$E_{s}$ [GPa]	$G_{s}$ [GPa]	$\nu_{s}$
Refai et al. [17]	Gyroid	0.1	3	110	41	0.34
Zhang et al. [19]	Diamond	0.3	5	190	73	0.30

**Table 2:** Homogenisation results for gyroid shape.

Source	$\bar{\rho }$	$\bar{E}$	$\bar{G}$	$\bar{\nu }$
Refai et al. [17]	0.1	0.032	0.035	1.000
FEM	0.1	0.032	0.035	1.000
MSG	0.1	0.032	0.035	1.000
FEM	0.5	0.249	0.293	0.896
MSG	0.5	0.249	0.293	0.896

**Table 3:** Homogenisation results for diamond shape.

Source	$\bar{\rho }$	$\bar{E}$	$\bar{G}$	$\bar{\nu }$
FEM	0.1	0.042	0.027	0.929
MSG	0.1	0.042	0.027	0.929
Zhang et al. FEM [19]	0.3	0.142	-	-
Zhang et al. EXP 1 [19]	0.3	0.135	-	-
Zhang et al. EXP 2 [19]	0.3	0.141	-	-
FEM	0.3	0.141	0.120	0.904
MSG	0.3	0.141	0.120	0.903
FEM	0.5	0.276	0.279	0.892
MSG	0.5	0.276	0.279	0.891

**Table 4:** Homogenisation results for PMY shape.

Source	$\bar{\rho }$	$\bar{E}$	$\bar{G}$	$\bar{\nu }$
FEM	0.1	0.031	0.036	0.992
MSG	0.1	0.031	0.036	0.991
FEM	0.5	0.289	0.314	0.912
MSG	0.5	0.289	0.314	0.911

**Table 5:** Homogenisation results for F-RD shape.

Source	$\bar{\rho }$	$\bar{E}$	$\bar{G}$	$\bar{\nu }$
FEM	0.1	0.042	0.023	0.748
MSG	0.1	0.041	0.023	0.741
FEM	0.5	0.330	0.295	0.826
MSG	0.5	0.330	0.296	0.826

## Data Availability

The original contributions presented in this study are included in the article. Further inquiries can be directed to the corresponding author.

## References

[1] Schwarz H.A. (1890). Gesammelte Mathematische Abhandlungen: Erster Band.

[2] Neovius E.R. (1883). Bestimmung Zweier Speciellen Periodischen Minimalflächen, auf Welchen Unendlich Viele Gerade Linien und Unendlich Viele Ebene Geodätische Linien Liegen.

[3] Schoen A.H. (1970). Infinite Periodic Minimal Surfaces Without Self-Intersections.

[4] Han L., Che S. (2018). An overview of materials with triply periodic minimal surfaces and related geometry: From biological structures to self-assembled systems. Adv. Mater..

[5] Ozden M.C., Simsek U., Ozdemir M., Gayir C.E., Sendur P. (2024). Innovative vibration control of triply periodic minimum surfaces lattice structures: A hybrid approach with constrained layer damping silicone–viscoelastic layer integration. Adv. Eng. Mater..

[6] Fu H., Huang X., Kaewunruen S. (2023). Experimental investigations into nonlinear dynamic behaviours of triply periodical minimal surface structures. Compos. Struct..

[7] Baraghani S., Abourahma J., Barani Z., Mohammadzadeh A., Sudhindra S., Lipatov A., Sinitskii A., Kargar F., Balandin A.A. (2021). Printed electronic devices with inks of TiS_3_ quasi-one-dimensional van der Waals material. ACS Appl. Mater. Interfaces.

[8] Honari P., Rahmatabadi D., Bayati A., Rassi E., Ghasemi I., Baniassadi M., Bodaghi M., Baghani M. (2025). Simultaneous 4D printing and in-situ foaming for tailoring shape memory behaviors in polylactic acid foams. Manuf. Lett..

[9] Zhang Y., Zhang J., Chen X., Yang W., Chen H., Che S., Han L. (2025). Mechanical properties of 3D-printed polymeric cellular structures based on bifurcating triply periodic minimal surfaces. Adv. Eng. Mater..

[10] Kashfi M., Nourbakhsh S.H., Amiripour A., Lim H.J. (2025). Constrained functionally graded gyroid structure for tunable energy absorption. Mater. Des..

[11] Al-Ketan O., Abu Al-Rub R.K. (2019). Multifunctional mechanical metamaterials based on triply periodic minimal surface lattices. Adv. Eng. Mater..

[12] Simsek U., Arslan T., Kavas B., Gayir C.E., Sendur P. (2021). Parametric studies on vibration characteristics of triply periodic minimum surface sandwich lattice structures. Int. J. Adv. Manuf. Technol..

[13] Zhang J., Chen X., Sun Y., Wang Y., Bai L. (2025). Vibration isolation and quasi-static compressive responses of curved gyroid metamaterials fabricated by selective laser sintering. Eng. Struct..

[14] Zou S., Mu Y., Pan B., Li G., Shao L., Du J., Jin Y. (2022). Mechanical and biological properties of enhanced porous scaffolds based on triply periodic minimal surfaces. Mater. Des..

[15] Abdulhadi H.S., Mian A. (2019). Effect of strut length and orientation on elastic mechanical response of modified body-centered cubic lattice structures. Proc. Inst. Mech. Eng. Part L J. Mater. Des. Appl..

[16] Zhang L., Feih S., Daynes S., Chang S., Wang M.Y., Wei J., Lu W.F. (2018). Energy absorption characteristics of metallic triply periodic minimal surface sheet structures under compressive loading. Addit. Manuf..

[17] Refai K., Montemurro M., Brugger C., Saintier N. (2020). Determination of the effective elastic properties of titanium lattice structures. Mech. Adv. Mater. Struct..

[18] Ramírez E.A., Béraud N., Montemurro M., Pourroy F., Villeneuve F. (2024). Effective elastic and strength properties of TPMS lattice structures by numerical homogenization. Mech. Adv. Mater. Struct..

[19] Zhang J., Xie S., Jing K., Wang H., Li T., He G. (2024). Study on isotropic design of triply periodic minimal surfaces structures under an elastic modulus compensation mechanism. Compos. Struct..

[20] Liu P., Liu A., Peng H., Tian L., Liu J., Lu L. (2021). Mechanical property profiles of microstructures via asymptotic homogenization. Comput. Graph..

[21] Yu W. (2016). A unified theory for constitutive modeling of composites. J. Mech. Mater. Struct..

[22] Yu W. (2019). Simplified formulation of Mechanics of Structure Genome. AIAA J..

[23] Chen Z., Xie Y.M., Wang Z., Li Q., Wu X., Zhou S. (2020). A comparison of fast Fourier transform-based homogenization method to asymptotic homogenization method. Compos. Struct..

[24] MathWorks (2025). MATLAB.

[25] Brakke K.A. (2023). The Surface Evolver.

[26] Khan K.A., Abu Al-Rub R.K. (2018). Modeling time and frequency domain viscoelastic behavior of architectured foams. J. Eng. Mech..

[27] Altair Engineering Inc (2025). Altair HyperMesh.

[28] Geuzaine C., Remacle J.-F. (2024). Gmsh.

[29] Al-Ketan O., Abu Al-Rub R.K. (2021). MSLattice: A free software for generating uniform and graded lattices based on triply periodic minimal surfaces. Mater. Des. Process. Commun..

[30] Marchais K., Chemisky Y., d’Esparbès R., Guevara M.R., Legerstee Y., sudeep5511 (2025). 3mah/microgen: V1.3.2.

[31] Yu W. (2016). An introduction to micromechanics. Appl. Mech. Mater..

[32] Barbero E.J. (2023). Finite Element Analysis of Composite Materials Using Abaqus^®^.

[33] Koutsawa Y., Tiem S., Yu W., Addiego F., Giunta G. (2017). A micromechanics approach for effective elastic properties of nano-composites with energetic surfaces/interfaces. Compos. Struct..

[34] Koutsawa Y., Karatrantos A.V., Yu W., Ruch D. (2018). A micromechanics approach for the effective thermal conductivity of composite materials with general linear imperfect interfaces. Compos. Struct..

[35] Dong G. 3D Homogenization of Cellular Materials. https://www.mathworks.com/matlabcentral/fileexchange/67457-3d-homogenization-of-cellular-materials.

